# Treatment barriers among young adults living with a substance use disorder in Tshwane, South Africa

**DOI:** 10.1186/s13011-022-00501-2

**Published:** 2022-11-19

**Authors:** Tichaenzana Nyashanu, Maretha Visser

**Affiliations:** Department of Psychology, University of Pretoria, Private Bag X20, Hatfield, 0028 South Africa

**Keywords:** Treatment barriers, Help-seeking, Substance use disorder, Young adults, Opioid substitution therapy

## Abstract

**Background:**

Despite increasing substance use globally, substance use treatment utilisation remains low. This study sought to explore and measure substance use treatment barriers among young adults in South Africa.

**Methods:**

The study was done in collaboration with the Community-Oriented Substance Use Programme run in Tshwane, South Africa**.** A mixed methods approach employing focus group discussions with key informants (*n* = 15), a survey with a random sample of people using substances and receiving treatment (*n* = 206), and individual semi-structured interviews (*n* = 15) was used. Descriptive statistics and thematic analysis were used to analyse data.

**Results:**

Contextual barriers seemed more prominent than attitudinal barriers in the South African context. Fragmented services, stigma-related factors, an information gap and lack of resources and support (contextual factors), perceived lack of treatment efficacy, privacy concerns, and denial and unreadiness to give up (attitudinal factors) were treatment barriers that emerged as themes in both quantitative and qualitative data. Culture and religion/spirituality emerged as an important barrier/facilitator theme in the qualitative data.

**Conclusion:**

Interventions need to embrace contextual factors such as culture, and more resources should be channelled towards substance use treatment. Multi-level stakeholder engagement is needed to minimise stigmatising behaviours from the community and to raise awareness of available treatment services. There is a need for strategies to integrate cultural factors, such as religion/spirituality and traditional healing, into treatment processes so that they complementarily work together with pharmacological treatments to improve health outcomes.

## Background

Similar to the international trend [1], the occurrence of substance use and substance use-related problems is rising in South Africa [2]. Alcohol and cannabis are the most commonly used substances in South Africa, and the use of illicit drugs such as heroin continues to rise [3]. In sub-Saharan Africa, South Africa is by far the largest market for illicit drugs [4], and it has one of the world’s highest opioid addiction rates and the fourth highest rate of drug offenses [5, 6]. Harmful substance use has been identified as a fuel or catalyst for crime, reduced productivity, unemployment, family violence and the escalation of chronic diseases such as HIV [7]. Despite the rampant use of substances and the associated negative consequences of substance use, there is a wide treatment gap as people using substances do not present themselves for treatment. This is one of the contributing factors to a wide treatment gap [8]. Previous research has attributed the treatment gap to a wide range of attitudinal and contextual barriers [9, 10]. In South Africa, complicated patient admission procedures, lack of awareness of treatment services, stigma, and perceived lack of need for treatment have been identified as prominent treatment barriers [11, 12]. There is a need for ongoing research on treatment barriers in South Africa since most of the research done is about a decade old and may not be relevant in the current context. This paper was written as part of a larger study on treatment barriers conducted by the authors in Tshwane, South Africa.

### Attitudinal barriers

The main attitudinal barriers identified in previous research are fear of stigma, privacy concerns and lack of perceived need for treatment [13]. Research has consistently shown that stigma is one of the most significant barriers to substance use treatment [14]. Typical of most disorders or illnesses that reach epidemic proportions (e.g., HIV and AIDS), the labelling of and discrimination against victims ultimately drive the victims to hide their illness to avoid discrimination, and this prevents them from seeking healthcare [14]. In South Africa, research has shown that stigma is generally more associated with people who use substances than with people who live with other mental disorders, and this is partly attributed to personal culpability associated with SUDs [15]. The use of heroin and heroin mixed with other substances is highly stigmatised, leading to marginalisation of people using substances, and rejection by their families and communities [16]. It has been observed that greater stigma is also attached to particular treatment methods, such as opioid substitution treatment (OST) [17], and other harm reduction interventions such as needle-and-syringe-exchange programmes.

Privacy concerns and lack of perceived need for treatment have been documented to hamper treatment utilisation among individuals living with SUDs [18]. Young adults are more likely than their older counterparts to report privacy concerns [18], as they want to avoid inquiry into and monitoring of their substance use [19]. Research in South Africa has found that those from low socio-economic backgrounds, where prevalence of substance use is often higher, are more likely not to know that they need help [20, 21]. They have learnt to justify, rationalise and normalise (as explained in cognitive dissonance theory) substance use as part of their lives and daily routine [22].

### Contextual barriers

Contextual barriers refer to structural factors that are perceived to preclude treatment utilisation. These include cultural factors, inadequate treatment facilities and personnel, fragmented services, and lack of information on available treatment services.

Culture is not exhaustively defined by language, ethnicity, nationality or race, but goes beyond that to focus on subcultures that are organised around shared values, beliefs, customs and traditions [23]. Cultural factors may mediate substance use and misuse behaviour, especially when substance use is related to cultural rituals and associated with strong masculinity [24]. Such cultural perceptions may ‘normalise’ substance use in these cultural groupings, which precludes people from perceiving a need for treatment [25]. Drug subcultures alienate themselves from mainstream society and establish social ties among people using substances. University or college students have been known to form such drug subcultures [26]. The Cape Flats drug subculture in South Africa is a prominent example [27] and is characterised by gangsterism and a strong culture of drug use and competition for control of a lucrative drug trade. One can argue that the norms and values of these cultural subgroups catalyse increased substance use and cause people using substances not to develop a sense of the need to regulate their use or seek treatment.

Global shortages of substance use treatment facilities and adequately trained healthcare personnel have negatively impacted on mental healthcare delivery, especially in low- and middle-income countries [28]. These shortages are largely due to a lack of public funding for mental health services [29]. In South Africa, certified training for addiction counsellors is limited and there is no provision for an addiction specialty for health professionals [30].

Fragmented services relate to characteristics of the healthcare system that impede treatment utilisation [31] such as flawed administrative practices, ineffectual laws and regulations, poor funding, poor data management systems, and poor staff training. In South Africa, slow registration of people using substances and long waiting lists have been identified as impediments to treatment utilisation [32]. The lack of competent and adequately trained mental healthcare practitioners who can productively engage and collaborate with patients dissuades people using substances from seeking treatment [33]. Research shows that people using substances prefer a collaborative relationship with practitioners to set out goals and negotiate a treatment plan [34].

Limited information about available treatment is one of main reasons why people using substances do not seek treatment [11]. Information about harm reduction strategies and treating opioid dependency using OST is especially limited [11, 21].

In order to address the paucity of rigorous research on barriers to substance use treatment in South Africa, this study aimed to explore and obtain an in-depth understanding of barriers to treatment among young adults living with SUDs, with a view to develop evidence-informed intervention strategies.

The objectives of the study were to:

1) explore what young people using substances perceive as barriers to treatment utilisation; 2) measure the relative strengths of different treatment barriers in impeding health services utilisation, using an instrument that has been adapted for the context; and 3) develop evidence-informed intervention strategies to enhance treatment options and accessibility of treatment for people using substances.

## Methods

### Setting

**Fig. 1:**
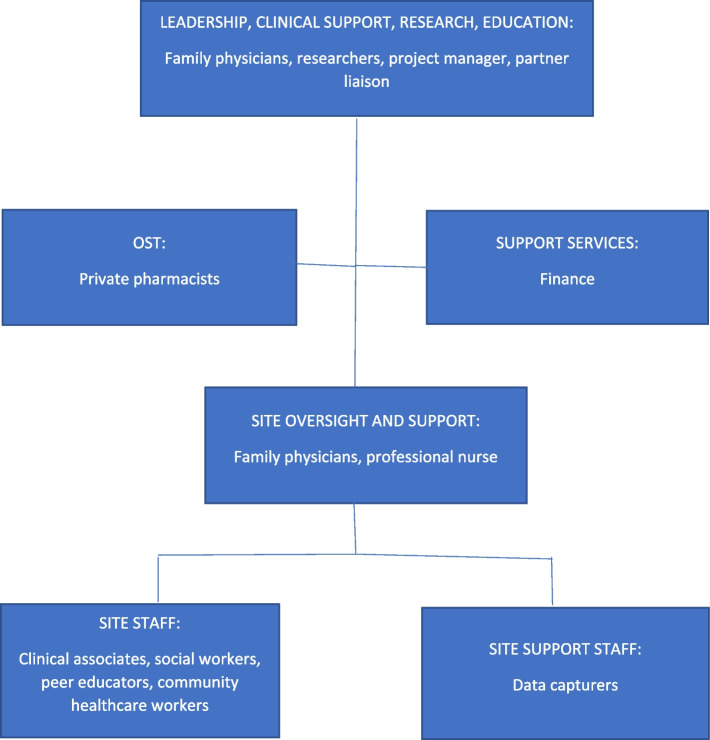
Staffing structures for COSUP [35]

The study was set in 17 sites in Tshwane, South Africa where the Community-Oriented Substance Use Programme (COSUP) is run. COSUP is a harm reduction initiative between the City of Tshwane and the University of Pretoria’s Department of Family Medicine. COSUP’s core service package provides screening, assessments, diagnosis, brief interventions, medical and counselling treatment services, and referrals of clients with problems related to substance use. Although COSUP provides treatment services for a wide range of substances, OST is one of the sought-after services because opioid use, particularly the use of heroin, is on the rise [2]. Figure 1 shows the COSUP staffing structure.

### Research approach

A mixed methods approach incorporating both the exploratory and explanatory sequential designs were employed in the research [34]. The research was three-phased, starting with focus group discussions (FGDs), followed by a questionnaire, and then by semi-structured interviews (SSI).

### Focus group discussions

FGDs are important in generating rich details of complex experiences on a defined topic [36].

#### Participants

Seventeen peer educators were purposively sampled as key informants and invited telephonically by the peer coordinator to participate in the FGDs. Peer educators in COSUP are individuals who formerly used substances, have been in treatment and have been abstinent for more than 6 months. They work under the supervision of a peer coordinator to link people using substances in the community to the COSUP services. Fifteen out of the seventeen peer educators (response rate of 88.2%) availed themselves for the research. Thirteen were males (86.7%) and two were females (13.3%). The mean age of the sample was 33.5 years (range = 29–44).

#### Data collection

Two sessions of face-to-face FGDs were conducted, with eight and seven participants respectively. FGDs were facilitated by the researcher and two research assistants who were fluent in the local languages. English language was used for the discussions as the participants were fairly fluent in English. In a few in stances, IsiZulu and Setswana were used by the participants and were translated by the research assistants. With the permission of the participants, the discussions were audio-recorded.

An example of a questions asked was:

From your individual experiences in using substances, and now as a peer educator, what are obstacles to treatment seeking? What are the reasons why young adults who use substances do not seek treatment?

#### Data analysis

The recorded data was transcribed verbatim. Translated sections were checked by the two multi-lingual research assistants. The data was separately coded (manually) by the researcher and two research assistants, using thematic analysis. Open coding was used to identify and analyse patterns of meaning in a dataset [25]. The researchers compared their coding and interpretation and reached consensus to assure trustworthiness of the data.

### Questionnaire

FGDs were used to adapt the 50-item BQ to try to overcome some of the weaknesses of the BQ which will be discussed under limitations of this study [37]. A high number of items were removed from the questionnaire due to redundancy. For example, questions on availability of medical insurance were removed as it was not relevant. From the FGDs, the researcher was able to add a few questions on culture to the questionnaire, because the theme of culture emerged more strongly later on in the SSIs. Examples of items added were: ‘Churches provide better services’ and ‘Our families encourage us to seek help from pastors and religious figures’.

Three independent experts confirmed the face validity of the questionnaire’s content and the researcher pilot tested the questionnaire with 12 peer educators, a subset of the study sample. Also, because the questionnaire was developed to measure characteristics in a different region of the world, some of the items became irrelevant in the South African context, especially with issues relating to culture.

#### Participants

The study population consisted of 512 young adults (aged between 18 and 29 years) receiving treatment from COSUP. A representative sample size, calculated using the survey sample size calculator method with a confidence level of 95% and a margin of error (confidence interval) of 5% [38], was 220. Random sampling, using random numbers (lottery method), was done from COSUP’s list of current clients. Eight recruited participants declined participation and six incomplete questionnaires were discarded, resulting in a final total of 206 respondents (response rate of 94%). The composition of the sample is given in Table 1.

**Table 1:** Demographic data of respondents

**Gender**	
Male	171 (83%)
Female	35 (17%)
**Age** (18–29 years)	mean = 26 years; SD = 3.52
**Race**	
Asian	2 (1%)
Black	151 (73.3%)
Coloured (mixed race)	46 (22.6%)
White	7 (3.4%)

#### Data collection

The self-report paper-and-pen questionnaire was administered to participants in small groups of not exceeding 20 participants at each of the COSUP sites. The answers were anonymised. Questions were answered on a 4-point Likert scale with options “strongly disagree - 0”, “disagree - 1”, “agree - 2” and “strongly agree - 3”.

#### Data analysis

Statistical Package for the Social Sciences (SPSS) version 27 was used for the analysis. In order to evaluate the characteristics of the questionnaire and to delete items with low reliability and factor loadings, the researcher performed exploratory factor analysis (EFA) using principal components extraction with Varimax rotation. Consequently, the questionnaire was reduced to 26 items loading on seven factors. The reliability of the questionnaire was investigated by means of internal consistency methods using Cronbach’s alpha.

Descriptive statistics were calculated for each barrier to show the relative strength of each. Additionally, the items were rank-ordered according to the frequency of being endorsed as a significant barrier within each theme/factor.

### Semi-structured interviews

#### Participants

A purposive sample (*n* = 15) of young adults (11 males and four females) who had completed the survey was invited to SSI. Half of the participants were sampled from inner city sites and half from peripheral township sites because the experiences of participants from the two sites seemed to differ.

#### Data collection

Participants were asked about the barriers to treatment they experienced while seeking help. They could give their views and explanations as well as additional information related to barriers identified through the quantitative data.

#### Data analysis

The procedure of data recording and transcribing was the same as that used for the FGDs. Thematic analysis [25] was used to analyse recurring themes in the data.

### Integration of results

Quantitative and qualitative results were compared, contrasted and integrated to develop an overall interpretation of the results [39]. The two qualitative approaches in this study complemented each other and are presented together to explain the quantitative data.

### Ethics approval and consent to participate

The study was granted ethical approval by the University of Pretoria’s Faculty of Humanities (Ref: 20795913 HUM012/0820). Written informed consent was received from all participants. Participation in the research was not remunerated.

## Results

As mentioned in the methods section, the qualitative phases (FGDs and SSIs) had 15 participants each, and the demographic characteristics of the qualitative samples were described in the methods section. The quantitative sample was representative of the race groupings and gender in the treatment sites. Notably, 79% of the COSUP clients were male and 21% were female.

The results are presented in the rank order of scales identified in the quantitative results (see Table 2). Themes identified from the qualitative data were integrated with the scales of the quantitative data where appropriate. This was done to understand how participants experienced the barriers to treatment.

**Table 2:** Barriers to treatment (*n* = 206)

Barriers	α	Mean	STD	Items	Frequencies		mean	STD
		Range 0–3			Disagree	Agree		
**Fragmented services** (single item)	–	2.51		Services are fragmented	60 (29.1%)	146 (70.9%)	2.51	1.180
**Stigma and discrimination in the community**	.756	2.26	0.681	The community looks down upon people using substances	37 (17.9%)	169 (82%)	2.30	0.887
				People blame us for our condition. They say it is our own fault	38 (18.4%)	168 (81.5%)	2.25	0.879
				People using substances are regarded as worthless	39 (18.9%)	167 (81.1%)	2.25	0.918
				The police abuse their power by ill-treating people using substances	42 (20.3%)	164 (79.6%)	2.24	0.889
**Information gap**	.697	1.92	0.845	The police lack information about treatment services so you can be unfairly arrested.	54 (26.2%)	152 (73.8%)	2.06	1.089
				I didn’t know where to go for help	58 (28.2%)	148 (71.8%)	1.94	1.025
				I didn’t know there is help available	74 (36%)	132 (64%)	1.77	1.096
**Labelling and rejection in the community**	.796	1.66	0.801	We feel not accepted across many different places and settings.	49 (23.8%)	157 (76.2%)	2.04	0.920
				I feared the shame and embarrassment of being called names.	74 (35.9%)	132 (64.1%)	1.82	1.092
				I feared losing my identity by being viewed as an outcast.	77 (37.4%)	129 (62.6%)	1.76	1.076
				I was afraid the community would isolate me	95 (46.1%)	111(53.9%)	1.50	1.112
				Healthcare workers mistreat people using substances	128(62.1%)	78 (37.9%)	1.17	1.181
**Lack of perceived treatment efficacy**	.661	1.50	0.759	Substance use treatment does not help	86 (41.7%)	120 (58.3%)	1.73	1.114
				Our families encourage us to seek help from pastors and religious leaders	88 (42.7%)	118 (57.3%)	1.65	1.137
				Churches provide better services	95 (46.1%)	111 (53.9%)	1.58	1.165
				I didn’t think treatment would do any good	101 (49%)	105 (51%)	1.52	1.200
				Treatment does not work	132(64.1%)	74 (35.9%)	1.04	1.207
**Privacy concerns**	.628	1.46	0.824	I thought I could handle it on my own and did not want people to know what I was going through.	81 (39.3%)	125 (60.7%)	1.69	1.137
				I didn’t want to talk about my personal life with other people.	94 (45.6%)	112 (54.4%)	1.24	1.101
**Lack of resources and support**	.681	1.43	0.860	I don’t get moral support from my family for treatment	76 (36.9)	130 (63.1%)	1.69	1.086
				Substance use healthcare sites are too few and far from where I stay	112(54.4%)	94 (45.6%)	1.37	1.152
				Substance use health care sites lack enough health care workers	117(56.8%)	89 (43.2%)	1.24	1.135
**Denial, not ready to give up**	.712	1.31	0.908	My substance use seemed fairly normal to me	107(51.9%)	99 (48.1%)	1.39	1.129
				I didn’t think I needed any help	116(56.3%)	90 (43.7%)	1.26	1.126
				I liked using substances and was not ready to give up	119(57.8%)	87 (42.2%)	1.26	1.164

Each of the scales will be discussed and qualitative data will be integrated to add some understanding of the experiences of the participants.

### Fragmented services

Fragmented services were the most endorsed item, with a mean of 2.51 (range 0–3). It is worth mentioning that this stand-alone item was significantly more important than other items measuring lack of resources. This item was part of the lack of resources factor, but was removed because of low internal consistency with the other items. In the qualitative data participants were critical about the treatment initiation process because it took long for a person in need of treatment to be initiated into the COSUP programme. They described the services as fragmented because of a lack of coordination and administrative problems to facilitate registration and treatment initiation:

- *So, they are made to wait for a long time for sessions where they must see doctors, they must see clinical associates, they must see the social workers. So, they won’t wait for that long. That’s why they won’t come.* (FGD 1, peer 8)
- *I would personally say for users not to come for treatment …. the reason lies with the way our system is working; it takes longer for them to get the medication.* (FGD 1, peer 8)

Participants noted with dismay that clients sometimes had to wait for six weeks before getting treatment:

- *They are too slow with the process. And sometimes you open a file; they take time to put you on treatment and people end up giving up.* (SSI, COSUP client 1)

### Stigma and discrimination from the community

Discrimination is a stigma-related barrier factor (mean = 2.26) that describes the blame and judgement the majority of the respondents (more than 80%) experienced in their communities. The qualitative data illustrates the judgement, negative perceptions and rejection the participants experience in all their interactions. The mark of ‘disgrace’ associated with the use of substances (stigma) culminates in the actual actions of prejudice, bias and intolerance (discrimination) towards people using substances [11], such as that displayed by the law enforcement. They ended up concealing their substance use and not seeking treatment:

- *The stigma is coming from everywhere, coming from peers, it’s coming from families, it’s coming from community leaders …. it’s just hitting them from everywhere.* (FGD 1, peer 4)
- *I would say stigma is one of the strongest reasons why most individuals don’t participate in the programme. They are in fear that they can’t be accepted to the community if they participate in a substance use treatment programme.* (FGD 1, peer 8)
- *The negative attitude towards people using substances bothers me a lot. People take us as if we are no longer able to think. They take us as if we are insane.* (SSI, COSUP client 2)
- *We are a vulnerable group of people whereby we are marginalised and we are used to being discriminated against everywhere we go, in health care and by the police. That’s why sometimes they don’t want to participate in substance use programmes like this.* (FGD 2, peer 4)

The community’s attitudes are reflected in the reaction of the police. The participants reported being subjected to indiscriminate harassment and abuse by the law enforcement agencies. Participants felt taken advantage of and disempowered:

- *Police, they are very rude. I don’t think they can really help places like COSUP because when they see a heroin addict, they see a criminal that must be locked up*. (SSI, COSUP client 7)
- *The fear is being instilled by the police because they deliberately arrest individuals for carrying methadone or syringes. They do not know what it is for.* (FGD 2, peer 5)

### Information gap

Information gap (mean = 1.92) was an important barrier factor as more than two-thirds of the participants were made aware of treatment services and where to get help only when they became COSUP clients. The qualitative data shows that most of the participants did not know where to get services, whereas others lacked information about the harm reduction treatment process:

- *I would say among 60% or 70% of young adults don’t know of treatment, there is not enough information about COSUP or about these institutions and where they are.* (FGD 1, peer 6)
- *For me to join COSUP I had suffered for a very long time, using substances and being unaware of treatment. I only found out about COSUP from one of my friends.* (FGD 1, peer 2)

Information about harm reduction and OST is limited, which creates many misperceptions:

- *There is lack of information on harm reduction.* … *they think that you have one bottle [methadone] and then you are healed. That’s what everyone believes and it’s not like that; it’s a continuous process.* (FGD 2, peer 4)

### Labelling and rejection in the community

This stigma-related factor was rated the fourth most significant barrier (mean = 1.66). One of the items, which reads “We feel not accepted across many different places and settings”, was endorsed by 76.2% of the participants. The qualitative data shows how participants were labelled with undesirable characteristics, were not been taken seriously and how this affected their self-esteem and identity. They felt like being viewed as misfits in society:

- *Stigma is the number one barrier whereby some of us, when people judge us, we no longer believe in ourselves.* (Focus Group 2, participant 4)
- *There is disrespect and discrimination and they call us with names such as nyaopes [heroin user].* (SSI, COSUP client 1)
- *I feel that many young people feel discouraged because whenever they seek help, they are not taken seriously. Health workers make jokes about their situations.* (FGD 2, peer 3)

### Perceived lack of treatment efficacy

Perception of the lack of efficacy of treatment was endorsed as a barrier to treatment by more than half of the participants (mean = 1.50). They were encouraged to rather use alternative services. In the qualitative data it came to the fore that especially a lack of information about harm reduction treatment may result in people thinking that treatment was not successful. The following narratives yielded qualitative data:

- *I often hear people saying that it doesn’t work for them. I don’t know if they are really using it the right way.* (SSI, COSUP client 3)
- *I think that the reason why young adults don’t participate in these programmes is that they feel that the medication isn’t working because most of the people have been on methadone for a year or two but still no change.* (FGD 2, peer 3)

### Privacy concerns

The privacy concern theme did not feature prominently as a barrier to treatment in this research (mean = 1.46). Some participants did not want to discuss their experiences with others. The item that read “I thought I could handle it on my own and did not want people to know what I was going through” was endorsed by 60.7% of the participants. In the qualitative data this theme was expressed as participants not wanting people to know they use substances:

- *They will be ashamed to be known to be substance users.* (FGD 2, peer 5)

### Lack of resources and support

Lack of resources and support (mean = 1.43) received lower endorsement in the quantitative data. The item that read “Substance use health care sites lack enough health care workers” was endorsed by 43.2% of the participants. In contrast, the qualitative data indicated more relevance of this theme. Participants expressed that there were too few treatment sites and not enough staff to attend to all the people that needs help:

- *It is true that there are too few COSUP sites and I think they need to have more sites so that people can get help.* (SSI, COSUP client 15)
- *COSUP sites lack enough workers. Sometimes they don’t have social workers that work full-time, or they are short of clinical associates.* (FGD 1, peer 4)

A large number of participants (63.1%) endorsed that they did not have the support of their families for treatment. The qualitative data shows how participants are rejected by family members and do not receive support in their treatment:

- *Their families no longer want to help with the journey to recovery.* (FGD 1, peer 4)
- *This is how most of these individuals get lost when they have everybody criticising them. Nobody is supporting, everybody is judging, not advising.* (FGD 2, peer 5)

### Denial, not ready to give up

This attitudinal barrier ranked as the least influential in this research (mean = 1.31). Each of the three items in the scale was endorsed by less than 50% of the participants. This could have been influenced by the fact that the participants were already in treatment and that their attitude of denial may have changed (see the section on limitations). Participants in the FGDs and SSIs highlighted denial and not being ready for treatment as important reasons not to seek treatment:

- *The reason is that they are in denial. It’s because they are not yet ready. They are still enjoying it. I remember while I was still smoking, I always had reasons why I smoked.* (SSI, COSUP client 10)
- *These programmes do help a lot but it all depends on a person because you can’t force someone if he doesn’t want to change; so, it all depends on a person. If you want something in life you must commit to it.* (FGD 1, peer 8)

### Culture

While not comparatively forming a significant part of the quantitative questionnaire, culture as a barrier/facilitator to seeking treatment emerged in the SSIs with COSUP clients. Whereas culture defines the attributes of a particular society at a particular time and place, traditional activities are more inclined towards reconnecting people with the past [40]. Some participants were of the opinion that beliefs in traditional medicine and healers (sangomas) were barriers to medical treatment, which is traditionally regarded as Western treatment. Religion/spirituality was also seen to counter the efforts of getting people who use substances into formal treatment. Families believe that religion, the church and prayer is the ultimate treatment for any problem:

- *Yes, religion can sometimes block us from getting help, because people believe that church is one of the first things that is needed for a person to be treated; prayer and attending church.* (SSI, COSUP client 12)
- *Cultural issues prevent one from seeking treatment, especially religion. I come from a very spiritual family, even this methadone they didn’t believe in it.* (SSI, COSUP client 3)
- On the other hand, some participants were of the opinion that religion and spirituality should be integrated into the harm reduction programme as religion is important in the recovery process:
- *Whatever you do, you can take medication but it is to your advantage and it helps you better if you are going to unite yourself with the church or calling on a higher power to help you to get over these SUDs.* (FGD 2, peer 5)

## Discussion

Several treatment barriers at personal (attitudinal) and contextual levels were identified. The findings of this study corroborate the findings of previous studies done in South Africa and other low-to-middle-income countries [41, 42] that contextual and structural barriers are of greater relevance in these countries than they are in high-income countries where attitudinal barriers tend to be more relevant.

Stigma (which is a contextual factor that influence individual behaviour) emerged as one of the most significant barriers to treatment-seeking among young adults living with SUDs. This finding is consistent with findings in earlier research done in South Africa [15] and internationally that stigma is one of the most significant treatment barriers facing people using substances [43]. This barrier theme forms a confluence with other systems in Bronfenbrenner’s socio-ecological model. For instance, Due to the negative labelling/perceived stigma of SUD in the community, people using substances anticipate rejection by their families and friends and doubt whether they will receive unprejudiced or appropriate healthcare from treatment centres. According to an ecological model [43], if many things influence processes through interrelationships, change in the environment through community awareness programmes to destigmatise SUD, among other things, may contribute to changing the availability of treatment and the attitude of the individual and family towards treatment seeking.

This study found that fragmented services and the provision of limited resources were also contextual factors that constrained accessibility and utilisation of treatment services. It is evident that the government policy on making mental health services (including substance use treatment) easily accessible and affordable by integrating these services into primary healthcare settings (e.g. South Africa’s 1997 White Paper Act on Health) has not been well implemented. COSUP services are part of primary healthcare efforts to increase accessibility and affordability of substance use services. However, as evidenced by participants’ comments that treatment facilities and healthcare workers are not enough, that services are fragmented and that there is a lack of information on available treatment, a gap in services still exists, which makes it difficult for people who use substances to receive treatment. To date, COSUP is South Africa’s only publicly funded substance use harm reduction [35]. This shows the extent of the lack of prioritisation of substance use treatment services.

This study’s findings confirm COSUP clients’ expectations of improved client handling approaches, which include stigma-free language usage and behaviour in professional and social settings. These expectations came to the fore in the qualitative data obtained.

According to the findings of this study, the influence of attitudinal barriers, which include privacy concerns and lack of perceived need of treatment are less impactful. Lack of mental health literacy in low-to-middle-income countries could be a factor contributing to denial by people using substances that they require treatment [44]. Furthermore, the lack of awareness about mental health issues may contribute to the stigmatisation of people with substance use-related problems, thereby prompting people using substances to hide their condition [14]. These findings are substantiated in related studies where HIV-related stigma has been linked to a lack of disclosing HIV status, heightened mental distress, and inability to establish new support systems [45].

### Recommendations

Creating greater community awareness of the effect of substance use-related problems and available treatment is a prerequisite to destigmatise substance use treatment in communities, law enforcement and policing, and the healthcare system. As indicated in this study, people using substances are unlikely to disclose their use or seek treatment because of stigma.

It is important to educate people about various treatment interventions, including OST which ranks as one of the most effective pharmacological interventions in the treatment of opioid dependence [46]. To prepare people using substances and those who support and live with them, systemic approaches, such as involvement of family/friends of the client in counselling, may encourage support for clients to aid the recovery process.

Based on findings of this study, there is a need for a high-level stakeholder engagement with police services so that they become partners and not adversaries in the substance use treatment drive. The over-surveillance of substance use treatment centres by the police has been found to deter patients from accessing help and treatment. Non-judgemental services can lead to greater participation in substance use treatment programmes. Improved client service skills of healthcare workers may increase the motivation of potential clients to seek help and treatment.

This study has indicated a need for cultural competence in mental health treatment, including the treatment of SUD. Because South Africa is a multicultural country, the integration of religious, traditional and Western approaches into treatment is important; therefore, calls have been made for collaboration between traditional health practitioners and primary health care services [47] to enhance cultural sensitivity in programmes such as COSUP. Substance use treatment facilities can supplement medical and psychosocial treatment services with spiritual/religious consultation services.

Peer-led community outreaches as implemented by the peer educators of COSUP are important to motivate people using substances to seek treatment as they can easily identify themselves with peers who are former users. Such outreaches may serve to make people using substances to see that change is possible, motivating them to seek treatment. Peer-led outreaches may also be essential to raise general awareness of substance use-related issues in communities.

### Limitations

In this study, data was mainly collected from people using substances who were already in treatment because it is difficult to access people using substances who may be in denial or have not yet utilised treatment services. This study recorded the experiences of COSUP clients already in treatment and of peer outreach workers working with people using substances but not yet receiving treatment. By virtue of their being in treatment, the participants (COSUP clients) had already overcome some treatment barriers. Young adults not receiving treatment may experience additional barriers or prioritise their experiences differently.

The theme of culture, which became one of the central themes in this study, had only a few questions/items in the quantitative phase of the study. This was largely because culture as a theme did not emerge prominently in the FGDs which were meant to be used to adapt the questionnaire, but emerged more prominently in the SSIs after the questionnaire had already been adapted and administered. The original 50-item questionnaire itself was not sensitive enough to the cultural aspects of the South African context because the questionnaire was not developed nor used locally, it had mostly been used in countries such as Mexico [37].

The scope of this study was limited to one province (i.e., Tshwane) in South Africa. Furthermore, the study collaborated with only one organisation in Tshwane that provides harm reduction treatment. These limitations could compromise the generalisability of the findings.

## Conclusion

Fragmented services, stigma, and information gap emerged as some of the most significant barriers to treatment utilisation. On the whole, contextual barriers seemed to exert greater influence on treatment utilisation, compared to attitudinal barriers. Qualitative data explored and revealed the relevance of culture as a barrier/facilitator to treatment, leading to suggestions on the possible importance of integrating medical treatment services with culturally relevant traditional and spiritual approaches to treatment to enhance treatment goals. Further, the results led the researcher to conclude that the findings could be used to inform policy and practice through a multi-level stakeholder engagement, for example, multi-sectoral and multi-level consultations geared especially towards addressing contextual barriers.

## Data Availability

The data that support the findings of this study are available from University of Pretoria but restrictions apply to the availability of these data, which were used under license for the current study, and so are not publicly available. Data are however available from the authors upon reasonable request and with permission of University of Pretoria.
